# A Single Bout of Exercise Increases the Expression of Glucose but not Fatty Acid Transporters in Skeletal Muscle of IL-6 KO Mice

**DOI:** 10.1007/s11745-012-3678-x

**Published:** 2012-05-24

**Authors:** B. Łukaszuk, I. Bialuk, J. Górski, M. Zajączkiewicz, M. M. Winnicka, A. Chabowski

**Affiliations:** 1 Department of Physiology, Medical University of Bialystok, ul. Mickiewicza 2C, 15-222 Bialystok, Poland; 2 Department of General and Experimental Pathology, Medical University of Bialystok, Bialystok, Poland

**Keywords:** IL-6, Skeletal muscle, Lipids, Glycogen, FA transporters, Glucose transporters

## Abstract

IL-6 is a biologically active cytokine released during exercise by contracting skeletal muscles. It appears to be highly involved in the regulation of muscles energy substrate utilization. Whether an ablation of IL-6 (IL-6 KO) in mice subjected to a single bout of exercise affects lipid and/or glucose metabolism is currently unknown. In the present study we examined fatty acid (FAT/CD36, FABPpm, FATP-1, FATP-4) as well as glucose (GLUT-1, GLUT-4) transporters expression in IL-6 KO mice. In addition, intramuscular glycogen and lipid content was also evaluated. The expression of all fatty acid transporters (FAT/CD36: +25 %; FATP-1: +31 %; FABPpm: +12.7 %; FATP-4: +7.2 %) was increased in muscles from IL-6 KO mice compared to wild type (WT) mice. Accordingly intramuscular lipid content was also increased in these muscles (FFA: +38 %; DAG: +36 % and TAG: +160 %). Interestingly, IL-6 deficiency had only minor effect on glucose transporters expression (GLUT-1: −4 %, and GLUT-4: −5.1 %), with no apparent difference in muscular glycogen content. A single bout of exercise increased the glucose transporters (GLUT-1: +8 %; GLUT-4: +15 %) as well as FA transporters (FAT/CD36: +13 %; FABPpm: +4.5 %; FATP: +2.5 %, FATP-4: +10 %) expression but only in WT skeletal muscles. In muscles from IL-6 KO mice exercise induced changes only in glucose (GLUT-1: +20 %; GLUT-4: +35 %) but not in the content of FA transporters. Concomitantly, IL-6 KO mice displayed shorter time toward exhaustion with more pronounced reductions in intramuscular lipid and glycogen content. We may speculate, that IL-6 deficiency provokes more pronounced glucose utilization over lipid oxidation during a single bout of exhausting exercise.

## Introduction

In the last few years, evidence has accumulated showing that interleukin-6 (IL-6) is an important molecule involved not only in immune regulation, hematopoiesis or inflammation but also in glucose and lipid metabolism [1]. IL-6 is synthesized by skeletal muscle and its secretion is markedly augmented during contractile activity [1, 2]. The resultant increase in IL-6 serum concentration positively correlates with increased glucose uptake and fatty acid (FA) oxidation in skeletal muscles. Likely both seem to be mediated by activation of AMPK (AMP-activated protein kinase) [1, 3, 4]. The activation of AMPK, which increases usage of energy substrates is commonly implicated in insulin sensitizing actions in skeletal muscles. However, this view has been recently challenged since serum IL-6 elevation was commonly observed in patients with metabolic disorders such as obesity and/or diabetes [5–7]. There is no evident explanation for this discrepancy. It might be speculated that IL-6 is also a potent activator of SOCS (suppressor of cytokine signaling) and thus increased activation of SOCS3 in skeletal muscles and liver leads to insulin resistance commonly observed in patients with obesity and diabetes [8].

At rest, skeletal muscles use both glucose and fatty acids (FA) as an equivalent energy source [9, 10]. However during exercise, the type of substrate used depends mainly on the intensity and the duration of exercise [2, 11]. It is generally well accepted that, with increased intensity of the exercise, there is a substantial enhancement in glucose utilization. Importantly, a crucial (but not exclusive) rate-limiting process in myocytes glucose utilization, involves transport of glucose across the sarcolemma. The process, that is specifically mediated by membrane glucose transporter proteins belonging to the facilitative GLUT/SLC2A family [12]. In contrary, fatty acids (FA), due to their hydrophobic nature, are able to rapidly traverse the lipid bilayer of the cell membrane by simple diffusion [13]. However, in addition to passive diffusion, a considerable evidence has accumulated to support the existence and importance of a protein-mediated FA transport [14–16]. In skeletal muscles a number of FA transporters have been identified, including (1) a family of fatty acid transport proteins (i.e. FATP-1,4), (2) plasma membrane associated fatty acid binding protein (FABPpm) and (3) fatty acid translocase (FAT/CD36) [17–19].

Given that IL-6 is an important muscle derived factor highly involved in myocyte energy substrate metabolism, we sought to investigate the effect of IL-6 ablation on glucose (GLUT-1, 4) and FA transporters (FAT/CD36, FABPpm, FATP-1,4) expression in skeletal muscle challenged by exercise. Additionally, intramuscular lipids and the glycogen contents were assessed. Aware of the age influence in IL-6 KO phenotype, we investigated the effect of a single bout of exhausting exercise in older mice (12 months).

## Materials and Methods

### Animals and Experimental Model

Male mice, wild type (C57B4/6J) and IL-6 KO (C57B4/6J IL-6^tml Kopf^−/−) were randomly allocated into two groups: sedentary and exercised till exhaustion (swimming). Until the day of experiment (12 months) mice were bred on site in an approved animal holding facility with free access to food and water. The study was conducted in accordance to guidelines of local ethic committee for animal care.

### Methods

#### Genotyping

IL-6 KO mice was tested using genomic DNA and PCR method to confirm deficiency of IL-6 gene as described elsewhere [20, 21]. Briefly, “Genomic mini” kit (A&A Biotechnology, Gdansk, Poland) was used to isolate DNA from mouse tails followed by PCR [applied custom made primers: F 5′-AAGTGCATCATCGTTGTTCATAC3′; R 5′-CCATCCAGTTGCCTTCTTG-3′ and commercially available DNA polymerase Taq “Marathon” (A&A Biotechnology, Gdansk, Poland)]. Subsequently DNA was separated on the 1 % agarose gel with ethidium bromide by means of electrophoresis. As a result we gained material from WT animals’ encompassed DNA fragments with size ca. 900 bp, whereas DNA fragments originate from knock-out mice (size about 1400 bp) additionally contained a fragment of neomycin cassette.

#### Exercise Protocol

Animals were used after 7 days of acclimatization to the laboratory conditions. They were maintained in a temperature-controlled environment (22 ± 1 °C) with a 12-h light–dark cycles beginning at 7 a.m. Experiments took place between 1.00 p.m. and 4.00 p.m. in the air-conditioned, sound-isolated room with the possibility of an accurate control of the light intensity. A single bout of exercise was carried out in a tank consisted of a circular, galvanized and painted in white steel pool (120 cm in diameter, 30 cm height) filled up to 26 cm depth with water maintained at 30 ± 1 °C. The mice were submitted to swimming exercise carrying load (lead fish sinkers, attached to the tail) corresponding to 5 % of their body weight. Animals were assessed to be fatigued when they remained under the water surface for 5 s [16].

#### Intramuscular FA (FAT/CD36, FABPpm, FATP-1,4), Glucose (GLUT-1, GLUT-4) Transporters and pAMPK/AMPK Protein Expression

The protein expression of FAT/CD36, FABPpm, FATP 1, FATP 4 and GLUT-1, GLUT-4 (50 μg) as well as AMPK and pAMPK (90 μg) was determined in muscle homogenates (soleus). Western blotting technique was used to detect the protein content as described by us and others [22, 23]. Briefly, the total protein content in each sample was determined by bicinchoninic acid method with BSA serving as a protein standard. Then, the proteins in each sample were separated using 10 % SDS-polyacrylamide gel electrophoresis and transferred to the nitrocellulose membrane. Equal protein concentrations were loaded in each lane as confirmed by Ponceau staining the blot membrane. In the next step membranes were immunoblotted with selected primary antibodies (FAT/CD36, FATP 1, FATP 4 (Abcam, EU) and GLUT-1, GLUT-4 and β-actin (Santa Cruz Biotechnology, US) as well as AMPK, pAMPK (Thr172) (Cell Signaling, US). Quantification of the selected protein content was achieved by densitometry [Optical Density (OD); Biorad, Poland]. The protein expression (FAT/CD36, FATP 1, FATP 4, GLUT-1 and GLUT-4) was related to β-actin and then to the control (WT sedentary or IL-6 KO sedentary, appropriately as indicated in the legends of figures) that was set to 100 %. Finally, each experimental group was expressed relatively (%) to the control. In addition, AMPK activation was expressed as pAMPK/AMPK ratio. Accordingly, Western Blots were made in triplicates.

#### Intramuscular Lipid Content

The mice were killed by cervical dislocation and immediately samples of soleus muscle were taken. Afterwards, the samples were rapidly cleaned from any visible non-muscle tissue, frozen in liquid nitrogen and finely powdered. Next the powder was transferred to a glass tube for consequent lipids extraction using the Folch et al. method [24] with modifications of van der Vusse et al. [25]. Gas liquid chromatography (Hewlett-Packard 5890 Series II gas chromatograph, HP-INNOWax capillary column) was used for identification and quantification of individual fatty acid methyl esters, whereas the total FFA, TAG, DAG concentration was estimated as the sum of particular fatty acid species content of the assessed fraction. The value was expressed as nanomoles per gram of wet tissue.

#### Intramuscular Glycogen Content

Soleus glycogen content was determined as described recently [23, 26]. Skeletal muscles were digested in 30 % KOH for 20 min and then, a spectrophotometric assay was used to qualify the content of free glucose. Glycogen level is expressed in μmol of glucose/g of wet tissue.

### Statistics

All data are expressed as means ± SEM. Statistical differences between groups were tested with analysis of variance (one way ANOVA) to determine the statistical significance, and appropriate post hoc test. Statistical significance was set at *p* ≤ 0.05.

## Results

Genotyping confirmed the deficiency of the functional IL-6 gene in the IL-6 KO mice as shown previously [20, 21]. IL-6 KO and wild type (WT) mice displayed no differences in body weight and serum glucose or FFA levels (data not shown, [21]).

### Effect of IL-6 KO Genotype on Basal Skeletal Muscle Fatty Acid Transporters Expression and Lipid Content

**Fig. 1:**
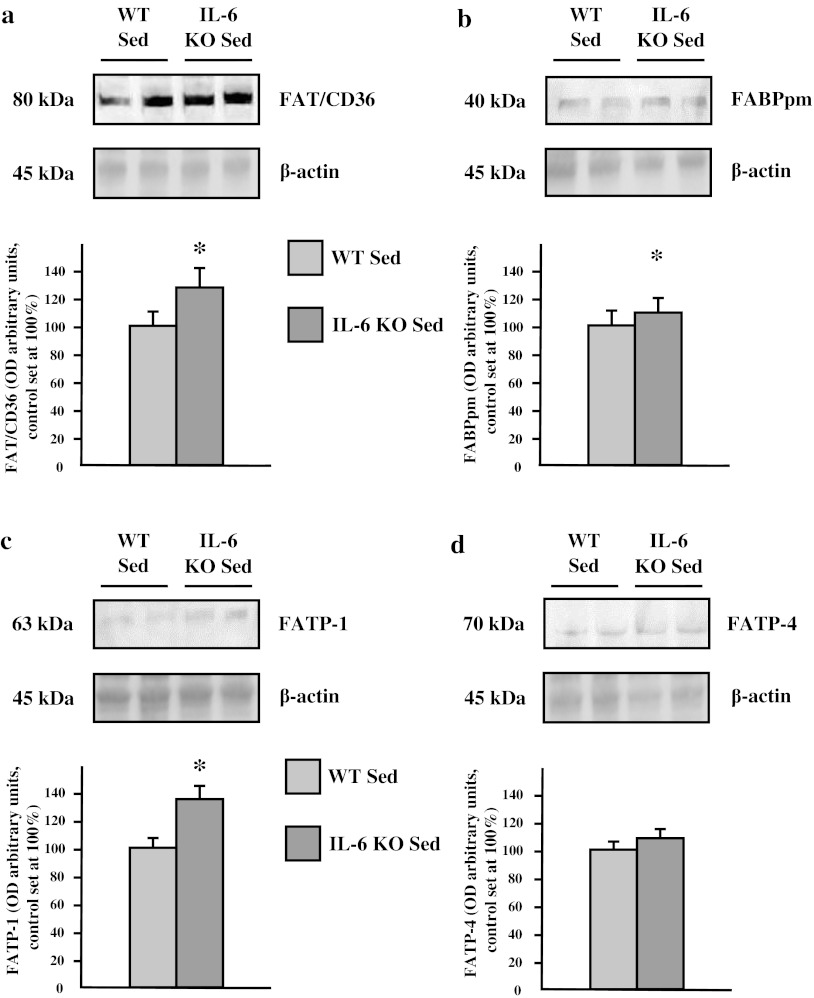
The effect of IL-6 deficiency on the expression of fatty acid transporters (**a** FAT/CD36, **b** FABPpm, **c** FATP-1, **d** FATP-4) in soleus muscle of sedentary mice. Representative Western Blots present a relative change in the protein expression related to β-actin content. (*n* = 8; *asterisk* WT Sed vs. IL-6 KO Sed, *P* < 0.05; *OD* optical density; control (WT Sed) set at 100 %)

**Fig. 2:**
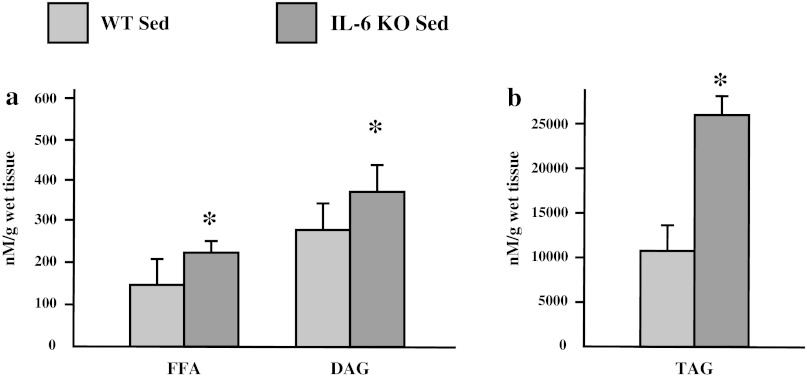
The effect of IL-6 deficiency on intramuscular lipid content (**a** FFA, DAG; **b** TAG), in soleus muscle tissue of sedentary mice. (*n* = 8; *asterisk* WT Sed vs. IL-6 KO Sed, *P* < 0.05, *FFA* free fatty acids, *DAG* diacylglycerols, *TAG* triacylglycerols)

In sedentary groups, the expression of fatty acid transporters (FAT/CD36: +25 %; FABPpm: +12.7 %; FATP-1: +31 %; *p* ≤ 0.05; FATP-4: +7.2 %; *p* > 0.05) was increased in muscles from IL-6 KO mice compared to wild type mice (Fig. 1a–d). Accordingly intramuscular lipid content was also increased in these muscles (FFA: +38 %; DAG: +36 % and TAG: +160 %; *p* ≤ 0.05) (Fig. 2a, b).

### Effect of IL-6 KO Genotype on Basal Skeletal Muscle Glucose Transporters Expression and Glycogen Content

**Fig. 3:**
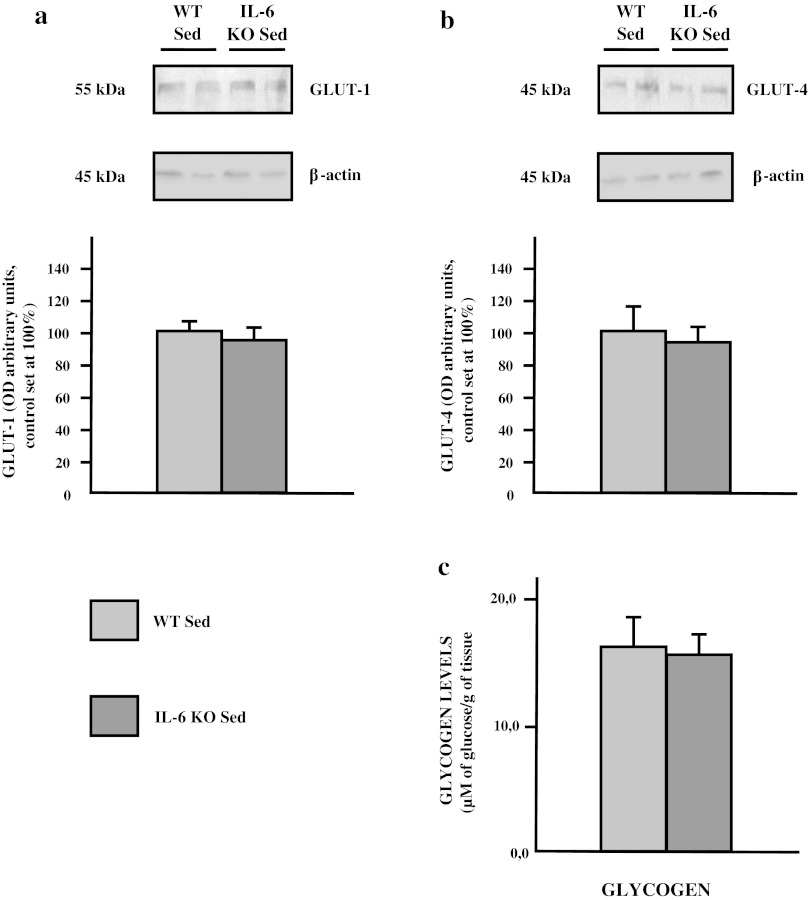
The effect of IL-6 deficiency on the expression of glucose transporters (**a** GLUT-1, **b** GLUT-4) in soleus muscle of sedentary mice. Representative Western Blots present a relative change in the protein expression related to β-actin content. (*n* = 8; *asterisk* WT Sed vs. IL-6 KO Sed, *P* < 0.05; *OD* optical density; control (WT Sed) set at 100 %)

Interestingly, IL-6 deficiency had only minor effect on: (1) glucose transporters expression (GLUT-1: −4 %, and GLUT-4: −5.1 %; *p* > 0.05; Fig. 3a, b) and (2) glycogen content in soleus muscle compared to control (−7 %; *p* > 0.05; Fig 3c).

### Effect of Single Bout of Exhausting Exercise and IL-6 KO Genotype on Skeletal Muscle Fatty Acid Transporters Expression and Lipid Content

**Fig. 4:**
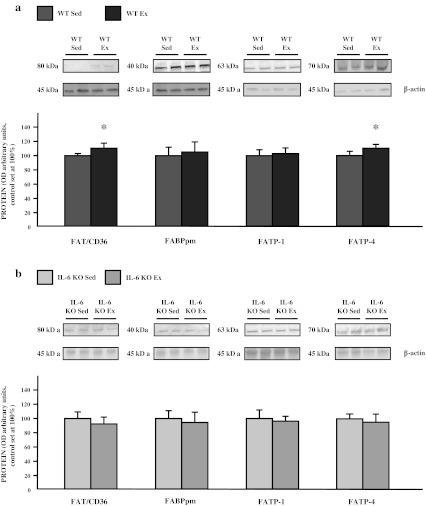
The effect of a single bout of exhausting exercise on the expression of fatty acid transporters (FAT/CD36, FABPpm, FATP-1, FATP-4) in soleus muscle of WT (**a**) and IL-6 KO mice (**b**). Representative Western Blots present a relative change in the protein expression related to β-actin content. (n = 8; *asterisk* **a** WT Sed vs. WT Ex, *P* < 0.05; control (WT Sed) set at 100 %); *asterisk* **b** IL-6 KO Sed vs. IL-6 KO Ex, control (IL-6 KO Sed) set at 100 %)

A single bout of exhausting exercise induced a small but significant increase in expression of both FAT/CD36 and FATP-4 (+13 and +10 %; *p* ≤ 0.05; Fig. 4a), with no apparent change in FABPpm and FATP-1 (+4.5 and +2.5 %; *p* > 0.05) in WT mice. No significant changes in FA transporters expression induced by exercise were observed in the IL-6 KO group (Fig. 4b).

**Fig. 5:**
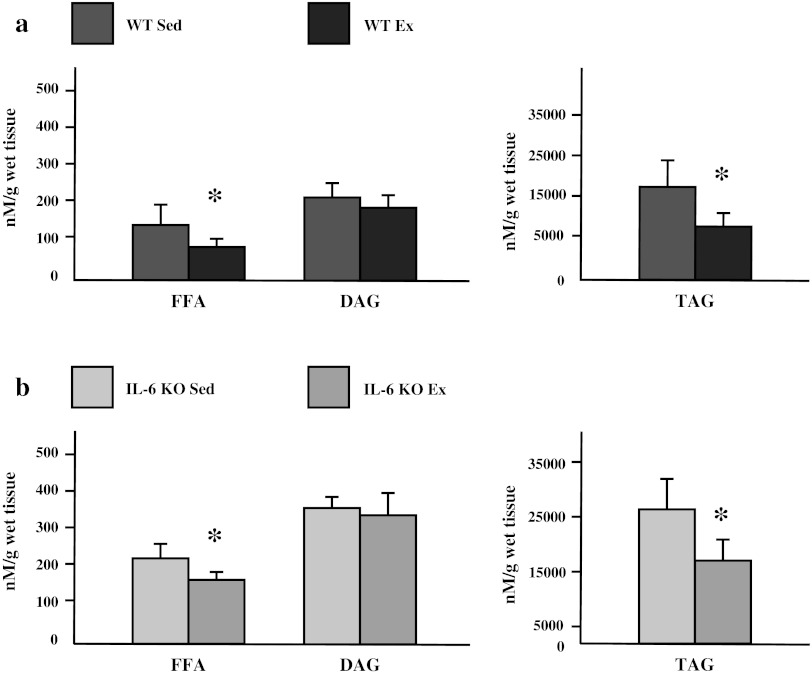
The effect of a single bout of exhausting exercise on the fatty acid content (FFA, DAG, TAG), in soleus muscle of WT (**a**) and IL-6 KO mice (**b**). (*n* = 8; *asterisk* WT vs. IL-6 KO, *P* < 0.05, *FFA* free fatty acids, *DAG* diacylglycerols, *TAG* triacylglycerols)

Applied exercise induced a significant reduction in intramuscular FFA and TAG content with more pronounced depletion in IL-6 KO group (−30 and −50 % in WT vs. −45 and −65 % in IL-6 KO group respectively; *p* ≤ 0.05; Fig. 5a, b). Apparently, not significant changes were observed in DAG fraction in both groups studied regardless the genotype.

### Effect of Single Bout of Exhausting Exercise and IL-6 KO Genotype on Skeletal Muscle Glucose Transporters Expression and Glycogen Content

**Fig. 6:**
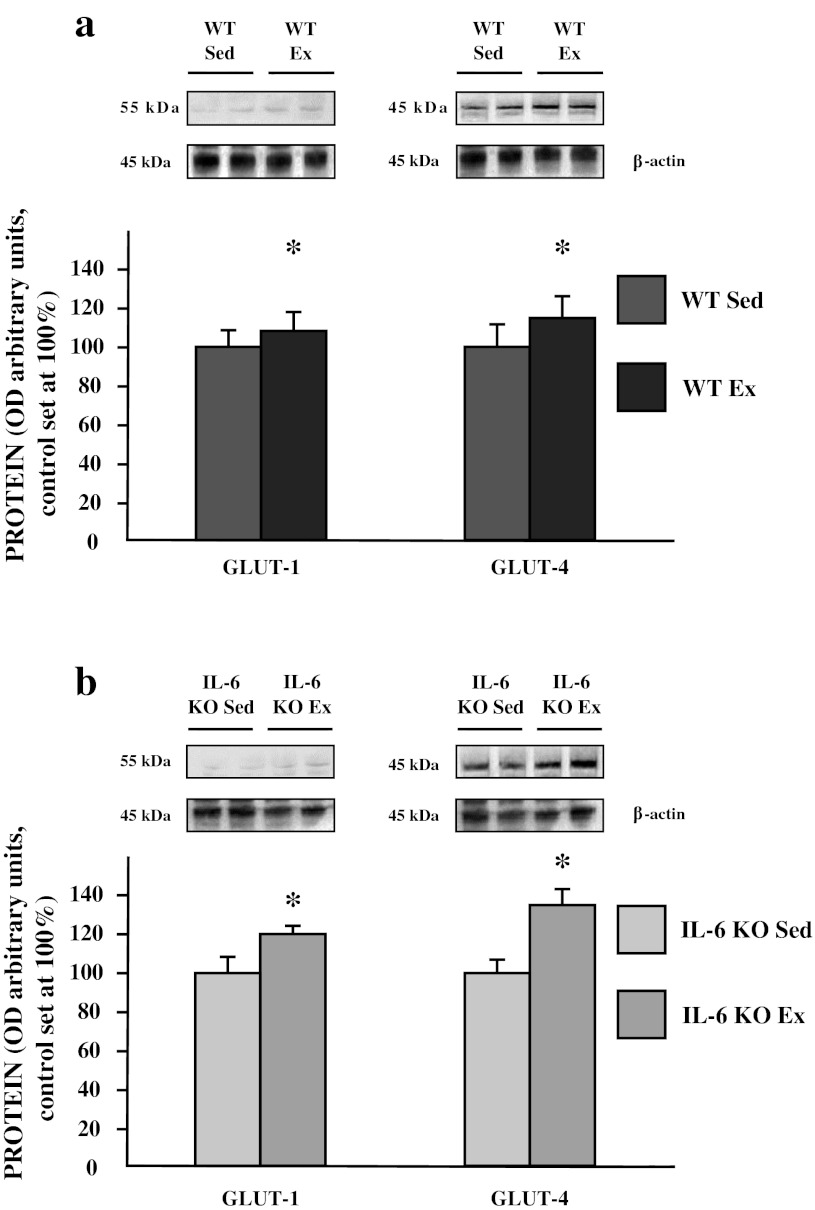
The effect of a single bout of exhausting exercise on the expression of glucose transporters: GLUT-1, GLUT-4 in soleus muscle of WT (**a**) and IL-6 KO mice (**b**). Representative Western Blots present a relative change in the protein expression related to β-actin content. (*n* = 8; *asterisk* **a** WT Sed vs. WT Ex, *P* < 0.05; control (WT Sed) set at 100 %); *asterisk* **b** IL-6 KO Sed vs IL-6 KO Ex, control (IL-6 KO Sed) set at 100 %)

Exercise induced a significant increase in glucose transporters (GLUT-1: +8 %, GLUT-4: +15 %; *p* ≤ 0.05; Fig. 6a) in WT mice, with more pronounced changes in IL-6 KO littermates (+20 and +35 %; *p* ≤ 0.05; Fig. 6b).

**Fig. 7:**
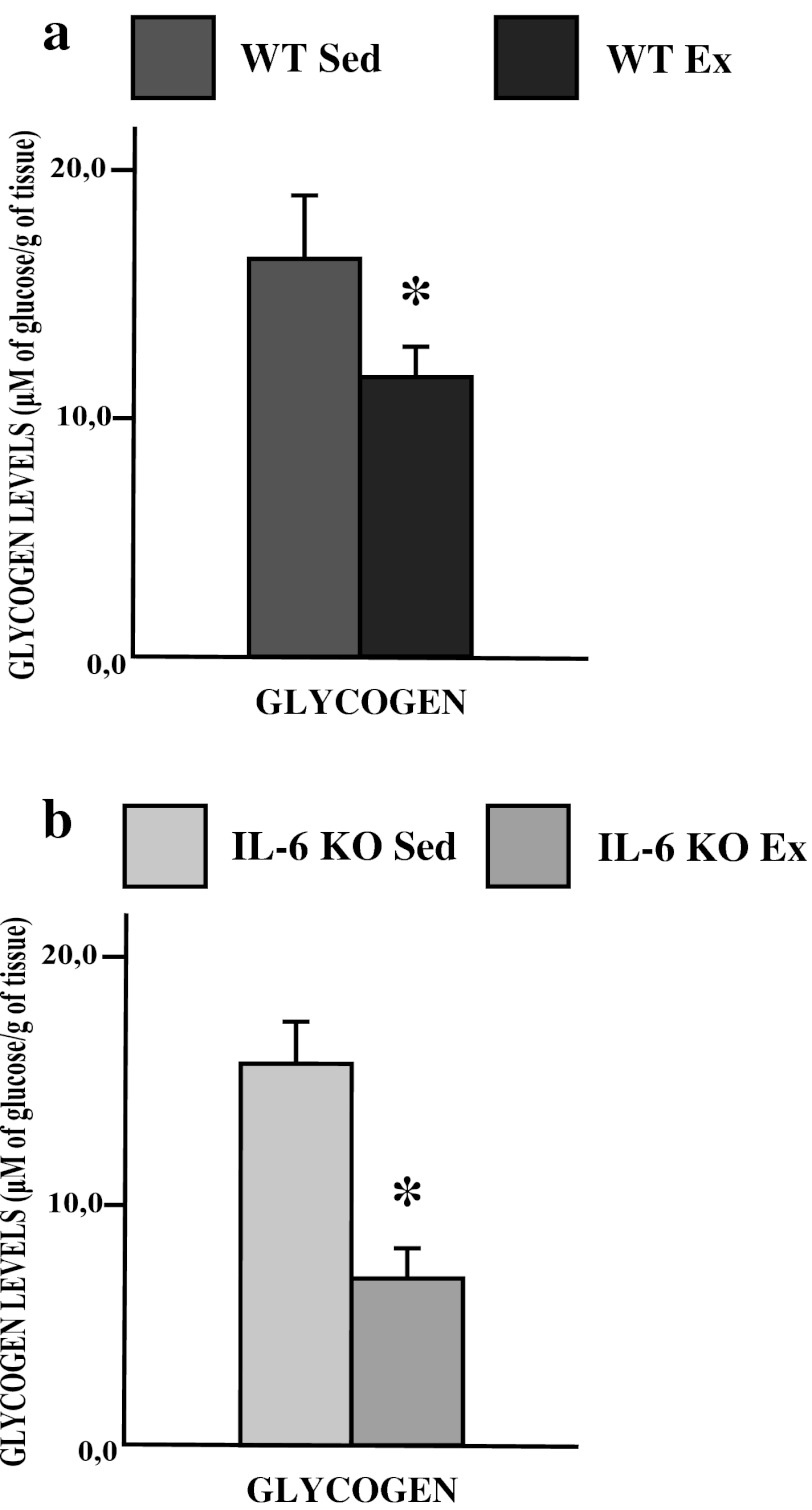
The effect of a single bout of exhausting exercise on the glycogen content (μM of glucose/g of tissue) in soleus muscle of WT (**a**) and IL-6 KO mice (**b**). (*n* = 8; *asterisk* **a** WT Sed vs. WT Ex and **b** IL-6 KO Sed vs. IL-6 KO Ex, *P* < 0.05)

In both WT and IL-6 KO mice, exhausting exercise induced significant reduction in soleus muscle glycogen level (−30 and 60 %, respectively; *p* ≤ 0.05 Fig. 7a, b).

### Effect of Single Bout of Exhausting Exercise and IL-6 KO Genotype on Skeletal Muscle AMPK Activation

**Fig. 8:**
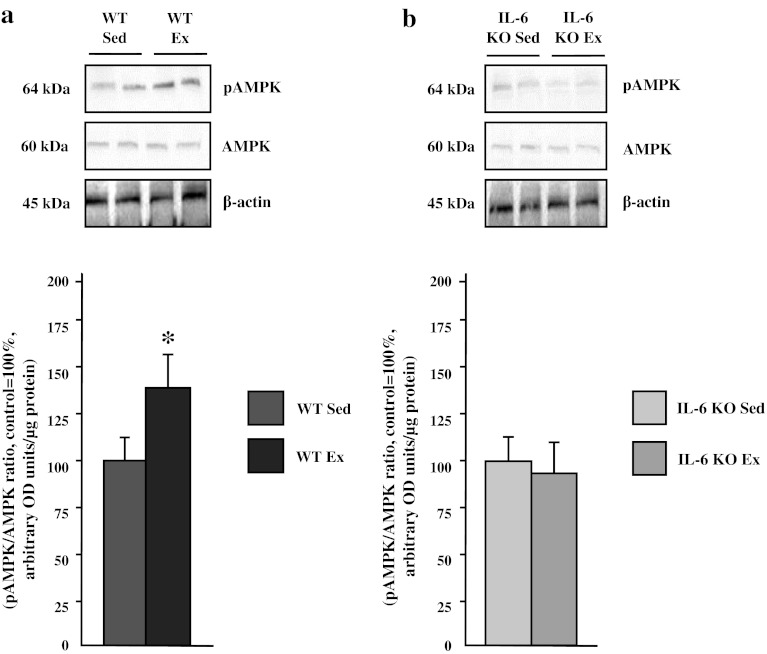
The effect of a single bout of exhausting exercise on pAMPK/AMPK protein expression in soleus muscle of WT (**a**) and IL-6 KO (**b**) mice. (*n* = 8; *double asterisks* **a** WT Sed vs. WT Ex, *P* < 0.05; control (WT Sed) set at 100 %); *asterisk* **b** IL-6 KO Sed vs. IL-6 KO Ex, control (IL-6 KO Sed) set at 100 %)

Exercise-induced AMPK activation as shown by increased the pAMPK/AMPK ratio (+40 %; *p* ≤ 0.05; Fig. 8), but only in WT mice, since we observed relatively lower (−10 %; *p* > 0.05; AMPK activation in IL-6 KO mice.

### Effect of Single Bout of Exhausting Exercise and IL-6 KO Genotype on Time Performance of Exercise

**Fig. 9:**
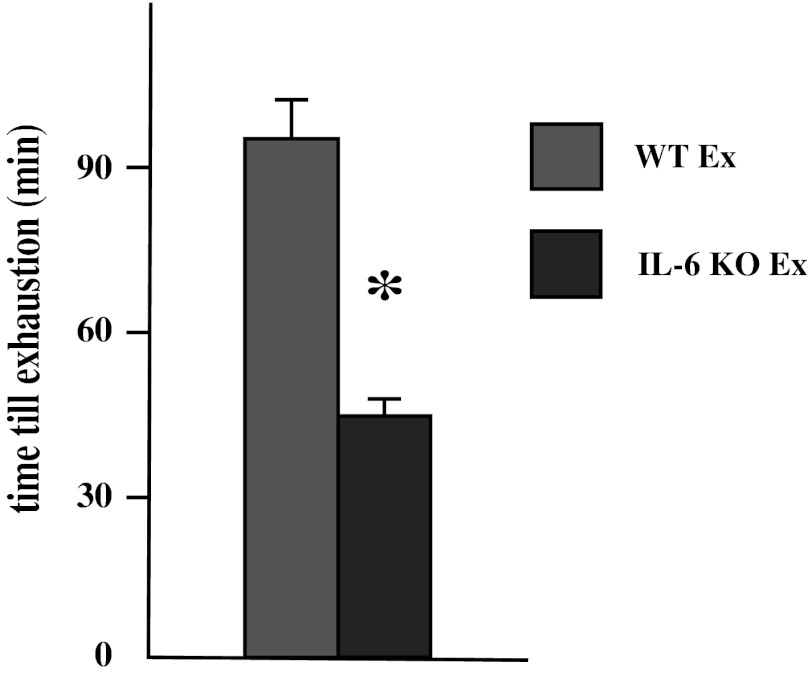
The effect of a single bout of exhausting exercise on the swimming duration time (*n* = 8; *asterisk* WT Ex vs. IL-6 KO Ex, *P* < 0.05)

IL-6 KO mice had a significantly reduced endurance capacity compared to WT mice. The swimming duration time for knock-out animals was much shorter (51 min), comparing with WT littermates (95 min) (*p* ≤ 0.05; Fig. 9).

## Discussion

The present study revealed that skeletal muscles from IL-6 deficient mice, when challenged by exercise, respond by: (1) increased expression of glucose (mainly GLUT-4), but not FA transporters and (2) increased reliance on intramuscular energy depots (−65 % TAG and −60 % glycogen reductions). These are accompanied by a lack of significant changes in the expression of AMPK kinase, when compared to WT littermates. Along with the findings, IL-6 KO mice displayed much shorter time till exhaustion during intense swimming. We confirmed also our earlier findings indicating a higher intramuscular lipid content (TAG, FFA, DAG) together with greater FA transport protein expression (FAT/CD36, FABPpm and FATP-1) in IL-6 KO mice [21]. However, despite intramuscular lipid accumulation and increased expression of FA transporters, IL-6 KO mice displayed no other features of obese phenotype (as shown in our previous study) [21, 27].

A growing amount of evidence implicates IL-6 as an important regulator of skeletal muscle metabolism. It seems likely that interleukin-6 exerts its effect on glucose uptake and FA oxidation in skeletal muscles via activation of AMPK (AMP-activated protein kinase) [4, 28]. Studies in vitro [29], ex vivo [30] and in vivo [31, 32] indicate a direct link between IL-6 and AMPK activation. The present study confirmed the existence of such a relations as evidenced by lower activation of AMPK during exercise in IL-6 KO mice comparing to WT littermates. Therefore, we may speculate also that, at rest, lack of functional IL-6 could significantly decreased basal AMPK activation, which in turn, resulted in decreased FA oxidation and subsequent accumulation of intramuscular lipids. This, together with an increased fatty acid transporters expression in skeletal muscle, was likely the cause of fatty acids accumulation in TAG and FFA fractions (present study and [21]). Additionally, functional IL-6 deficiency when challenged by exercise, induced a smaller reduction in intramuscular lipids that, again, can be attributed to the lack of significant AMPK activation (and AMPK-related FA oxidation). Based on the above, we may speculate that IL-6 deficiency diminishes the AMPK activation during exercise and it seems likely, that exercise-induced activation of AMPK does not directly require IL-6 [33]. During exercise IL-6 serum levels may be increased as much as 100-fold) [34, 35] and in WT mice greater AMPK activation results not only in a response to the increased ratio of AMP/ATP but is additionally triggered by an IL-6 dependent pathway [36]. An open question remains, whether these effects are additive in skeletal muscles. Our findings suggest such a possibility, since, in WT mice, exhausting exercise induced not only a depletion of endogenous energy stores (a reduction in TAG and glycogen), but also induced glucose and fatty acid transporters expression, exacerbating exogenous energy substrates flux into the muscles. It is well known that AMPK activation results in robust energy provision and many studies have shown both: increased FA and glucose oxidation as well as enhanced FA and glucose transporters expression [14, 37, 38]. The present study suggests that with IL-6 deficiency, at least when AMPK activation is diminished, FA flux and subsequent FA oxidation are reduced. Concomitantly, this genetic lack of functional IL-6, associated with diminished exercise-induced AMPK activity, resulted in the increased expression of GLUT-4. It leaves an open discussion for other factors that may also be involved in favoring glucose metabolism over FA oxidation. Furthermore, IL-6 deficient animals exhibited diminished capacity for endurance performance. Likely, it is related to the respiratory exchange ratio (RER), since others reported that during exercise IL-6 KO mice reveal decreased exercise capacity [39]. Likewise, decreased AMPK activation (present study and [39]) could contribute to the shorter time till exhaustion as it was shown in mice expressing a dominant negative AMPK [40].

Along with this idea are studies showing that IL-6 may play a significant role in angiogenesis processes [41]. Thus, IL-6 deficiency may cause a decreased oxygen supply that contributes to the decreased lipid utilization [42]. It has also been suggested that IL-6 released from contracting skeletal muscles stimulates the release of glucose and FA from the liver and adipose tissue [1, 2] and lack of functional IL-6 may result in a reduced plasma energy substrate availability. We may speculate this may account for greater reliance on intramuscular energy depots observed in IL-6 KO mice (a higher reduction of intramuscular glycogen and TAG). Nevertheless, the exact mechanism responsible for decreased endurance capacity in IL-6 deficient mice needs to be clarified.

Summing up, the present study has provided several novel findings. We confirmed greater expression of FA transport proteins and accumulation of intramuscular lipids in soleus from IL-6 KO sedentary mice. We found also that IL-6 deficiency results in shorter time till exhaustion accompanied by pronounced intramuscular glycogen depletion, with substantial changes in glucose but not fatty acid transporters expression.

## Abbreviations

AMPK: AMP-activated protein kinase
DAG: Diacylglycerols
DNA: Deoxyribonucleic acid
FA: Fatty acids
FABPpm: Plasma membrane associated fatty acid binding protein
FAT/CD36: Fatty acid translocase
FATP-1: Fatty acid transport protein isoform 1
FATP-4: Fatty acid transport protein isoform 4
FFA: Free fatty acids
GLUT-1: Glucose transporter isoform 1
GLUT-4: Glucose transporter isoform 2
IL-6: Interleukin-6
KO: Knock-out animals
PCR: Polymerase chain reaction
SEM: Standard error of the mean
SOCS: Suppressor of cytokine signaling
TAG: Triacylglycerols
WT: Wild type animals
